# Supplementary material for: Chemical instability at chalcogenide surfaces impacts chalcopyrite devices well beyond the surface
Source: Nat Commun. 2020 Jul 20;11:3634. doi: 10.1038/s41467-020-17434-8 (PMC7371883; doi:10.1038/s41467-020-17434-8)
Supplement: Supplementary file 1 — Supplementary Information [file 41467_2020_17434_MOESM1_ESM.pdf]

Supplementary Information

Chemical instability at chalcogenide surfaces impacts chalcopyrite devices well beyond the surface

Colombara, D. et al.

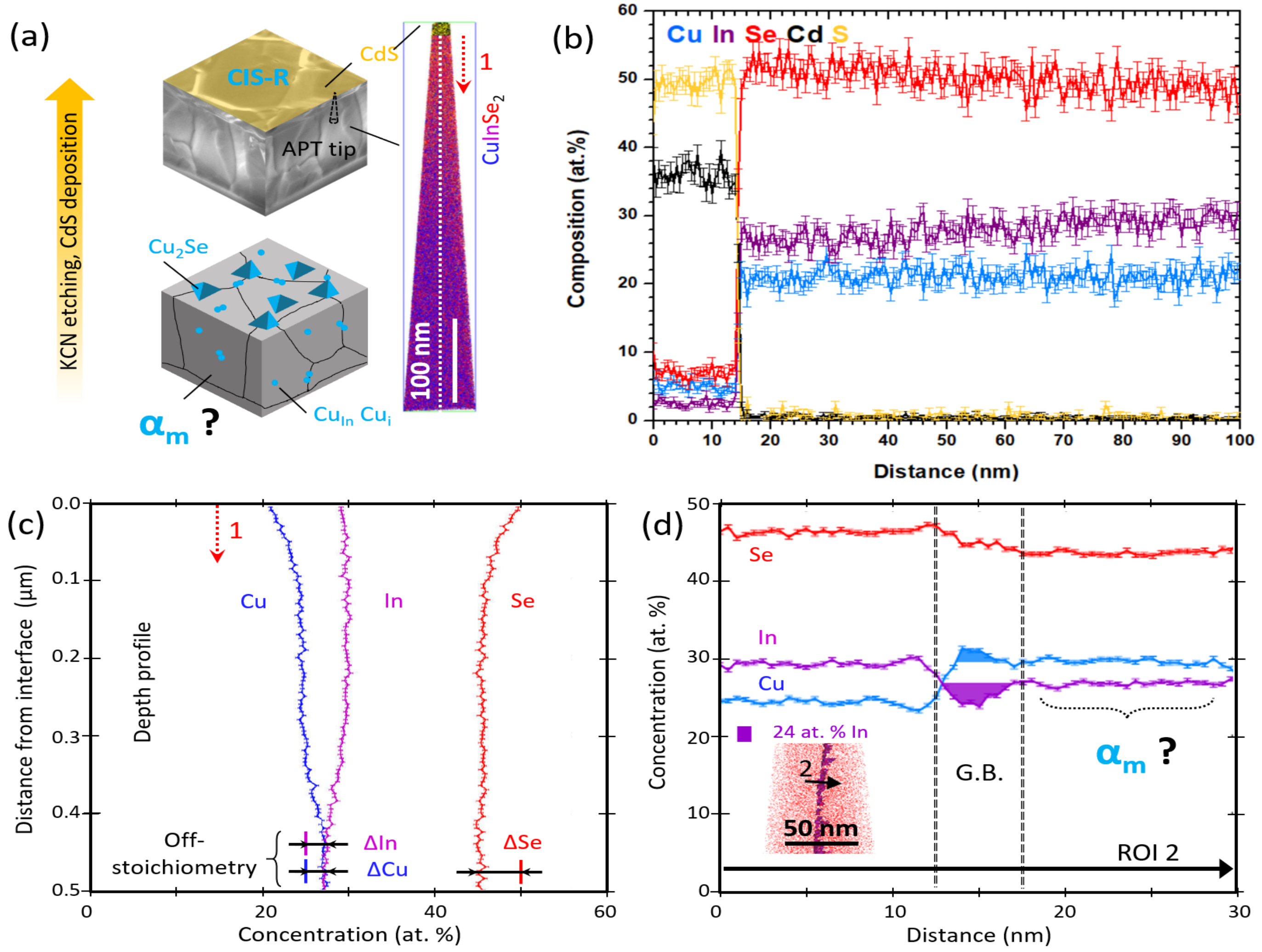

Supplementary Figure 1 – Atom probe tomography of Cu-rich CIS. (a) Schematic description of the Cu-rich sample preparation before APT analysis consisting of removal of the excess Cu_2Se phase by KCN surface etching and CdS coating by chemical bath deposition. Elemental distribution map of Cu (blue), In (magenta) and Se (red). **(b)** Compositional profile in the vicinity of the CIS/CdS interface. **(c)** Compositional depth profile of an equivalent sample revealing substantial Cu depletion from the interface down to ca. 400 nm compared to unetched CIS-S. **(d)** Compositional profile across ROI 2, a grain boundary (GB) of another Cu-rich specimen (cf. inset where the GB is marked by a 24 at % In iso-surface), revealing a portion with Cu concentration far exceeding stoichiometry (dotted curly bracket).

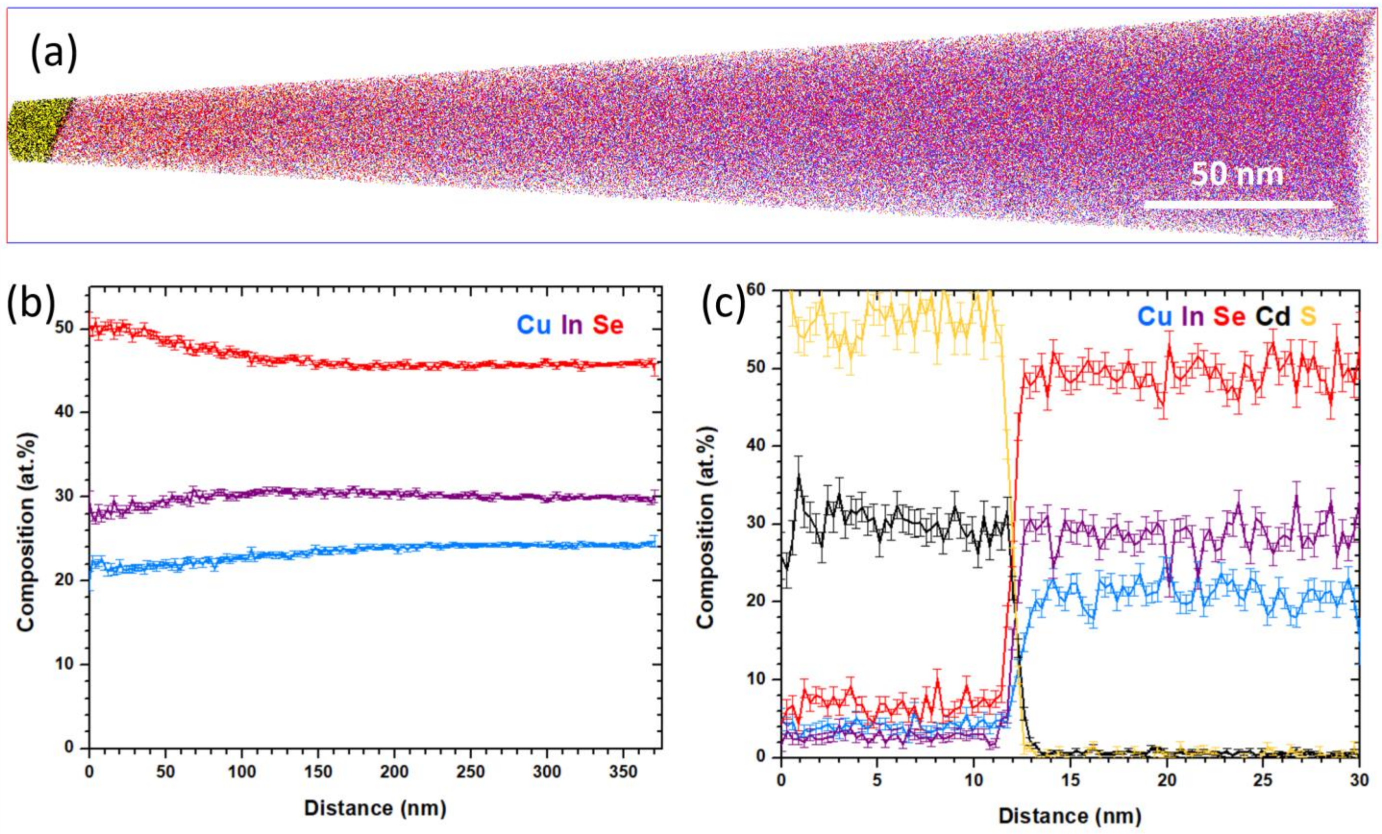

Supplementary Figure 2 – Atom probe tomography of Cu-poor CIS. (a) Atom probe tomography of a Cu-poor CIS layer (note that the sample was grown via the three-stage process) subject to KCN etching and CdS deposition by CBD. **(b)** Corresponding concentration profile along the entire dataset. **(c)** Concentration profile in the vicinity of the CIS/CdS interface.

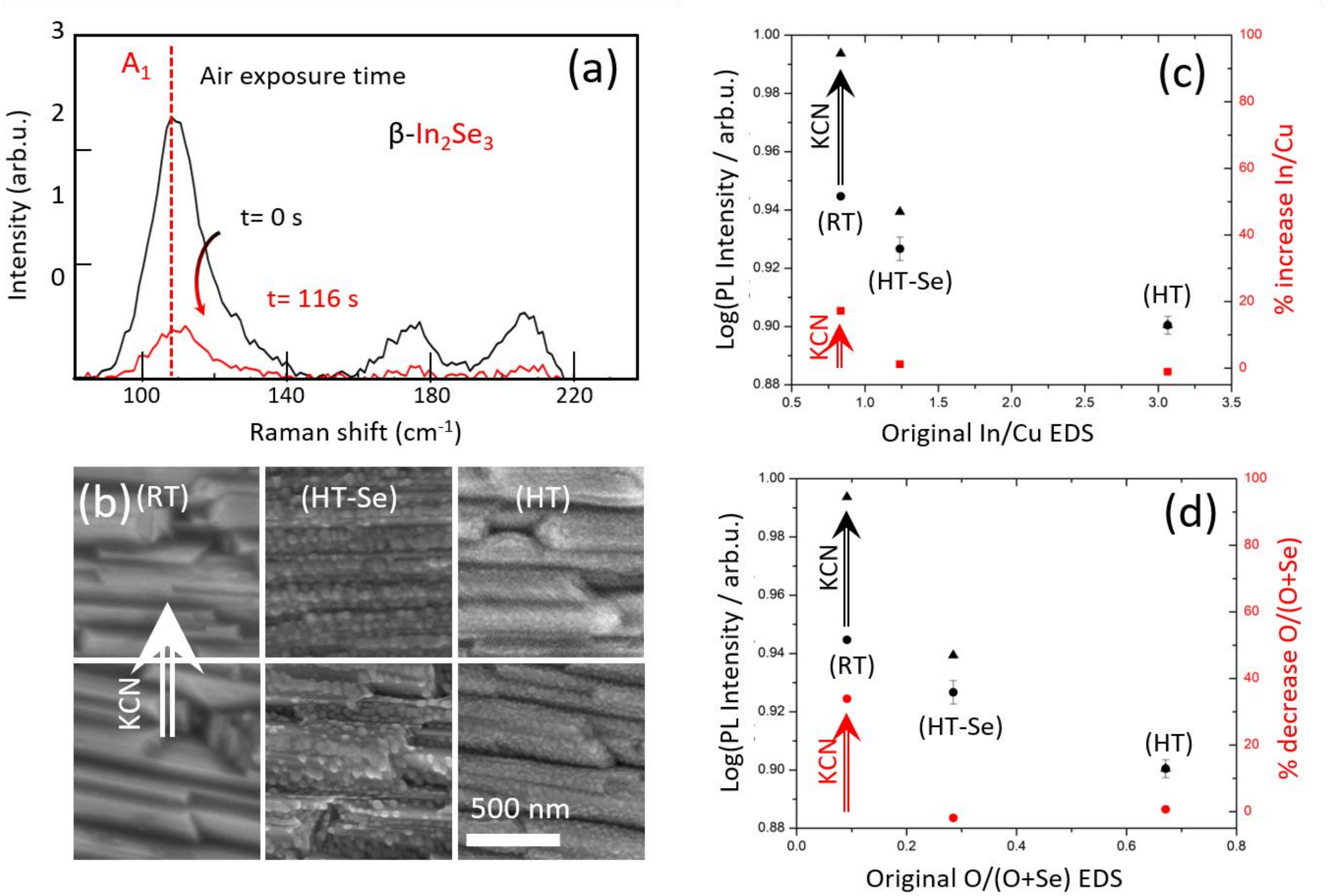

Supplementary Figure 3 – **Oxidation of In_2Se_3 and CIS bulk and surfaces. (a)** Raman spectra of a thin β-In_2Se_3 film before (black) and after (red) exposure to air under the 532 nm laser beam, revealing a 6-fold decrease of the A_1 interlayer vibrational mode over a timeframe of 116 seconds. **(b)** SEM micrographs of epitaxial Cu-poor CIS films in their oxidised state (bottom images) and after KCN etching (top images). **(c)** Combined PL intensity (black data and ordinate) and increase of In/Cu EDS ratio (red data and ordinate) versus the original In/Cu EDS ratio of the oxidised films before and after KCN etching. **(d)** Combined PL intensity (black data) and decrease of O/(O+Se) EDS ratio (red data) versus the original O/(O+Se) EDS ratio of the oxidised films before and after KCN etching.

	Cu-rich	Cu-poor	ΔΔG / eV
**III_2O_3 partial**	$2Cu[III_{(1-y)}(Cu_{III})_y(V_{III})_z]Se_{2(s)} + \frac{3x}{2}O_{2(g)} \rightarrow xIII_2O_{3(s)} + 2Cu[III_{(1-y-x)}(Cu_{III})_y(V_{III})_{z+x}]Se_{2(s)}$ (04)	$2[Cu_{(1-y-z)}(III_{Cu})_y(V_{Cu})_z]IIISe_{2(s)} + \frac{3x}{2}O_{2(g)} \rightarrow xIII_2O_{3(s)} + 2[Cu_{(1-y-z)}(III_{Cu})_{y-x}(V_{Cu})_{z+x}]IIISe_{2(s)}$ (05)	
**ΔG / eV**	$xG[III_2O_{3(s)}] + 2xG[(V_{III})] - \frac{3x}{2}G[O_{2(g)}]$ *4.50* 4.48 6.00	$xG[III_2O_{3(s)}] + 2xG[(V_{Cu})] - \frac{3x}{2}G[O_{2(g)}] - 2xG[(III_{Cu})]$ *1.25-0.25=1.0* 0.76-0.82=-0.06 2.94-1.42=1.52	*+3.50* +4.54 +4.48
**Cu_2Se partial**	$2Cu[III_{(1-y)}(Cu_{III})_y(V_{III})_z]Se_{2(s)} \rightarrow xCu_2Se_{(s)} + 2Cu[III_{(1-y)}(Cu_{III})_{y-x}(V_{III})_{z+x}][Se_{(1-x/4)}(V_{Se})_{x/4}]_{2(s)}$ (06)	$2[Cu_{(1-y-z)}(III_{Cu})_y(V_{Cu})_z]IIISe_{2(s)} \rightarrow xCu_2Se_{(s)} + 2[Cu_{(1-x-y-z)}(III_{Cu})_y(V_{Cu})_{z+x}]III[Se_{(1-x/4)}(V_{Se})_{x/4}]_{2(s)}$ (07)	
**ΔG / eV**	$xG[Cu_2Se_{(s)}] + 2xG[(V_{III})] + xG[(V_{Se})] - 2xG[(Cu_{III})]$ *4.5+2-1.1=5.4* 4.48+2.00-1.30=5.18 6.00+2.47-1.76=6.71	$xG[Cu_2Se_{(s)}] + 2xG[(V_{Cu})] + xG[(V_{Se})]$ *1.25+2.00=3.25* 0.76+2.00=2.76 1.88+2.47=4.35	*+2.15* +2.42 +2.36
**Cu_2Se path (i)**	$2[(Cu_{Cu})_{(1-y)}(Cu_{i.})_y]\,IIISe_{2(s)} \rightarrow xCu_2Se_{(s)} + 2[(Cu_{Cu})_{(1-y)}(Cu_{i.})_{y-x}][Se_{(1-x/4)}(V_{Se})_{x/4}]_{2(s)}$ (08)	———	
**ΔG / eV**	$xG[Cu_2Se_{(s)}] + xG[(V_{Se})] - 2xG[(Cu_{i.})]$ *2.00-1.00=1.00* 2.00-2.00=0.00 2.47-2.12=0.35		*-2.25* -2.76 -4.00
	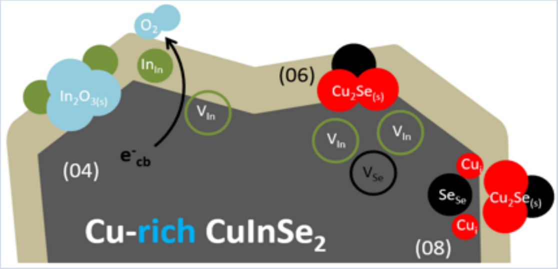	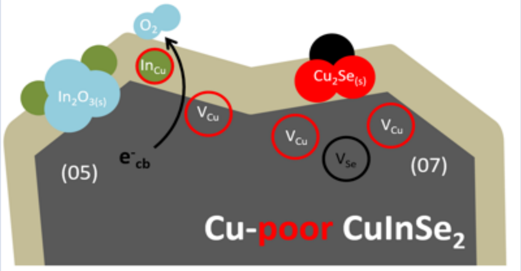	

Supplementary Figure 4 – CIS Point defect reactions. CIS and CGS partial oxidation reactions expressed in terms of point defects and corresponding Gibbs free energies at 0 K. Note that comparison of the values is only possible for the rightmost column ΔΔG representing the Gibbs energy difference between Cu-rich and Cu-poor reactions. The energetics of point defect formation under Cu-rich and Cu-poor compositions (points B and C, respectively) are taken from Malitckaya et al. [1] (italic values) and Pohl and Albe [2] (green values for CGS). A schematic representation of the reactions is shown in the drawings for the two cases.

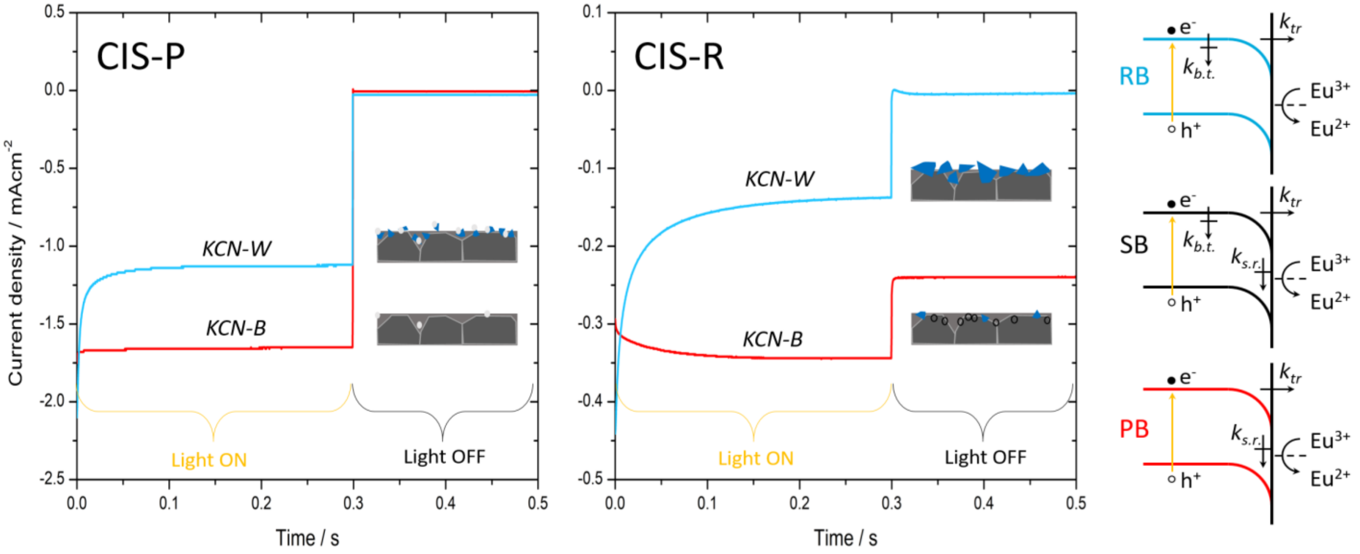

Supplementary Figure 5 – Photoelectrochemical (PEC) analyses. PEC analysis of CIS-P and CIS-R films subject to KCN-W and KCN-B etching. Note the large difference in the ordinate axis scale between the two: the photocurrent magnitude of CIS-R is approximately 10 % of that of CIS-P, pointing to a larger bulk recombination and/or semiconductor/electrolyte interface recombination in CIS-R compared to CIS-P.

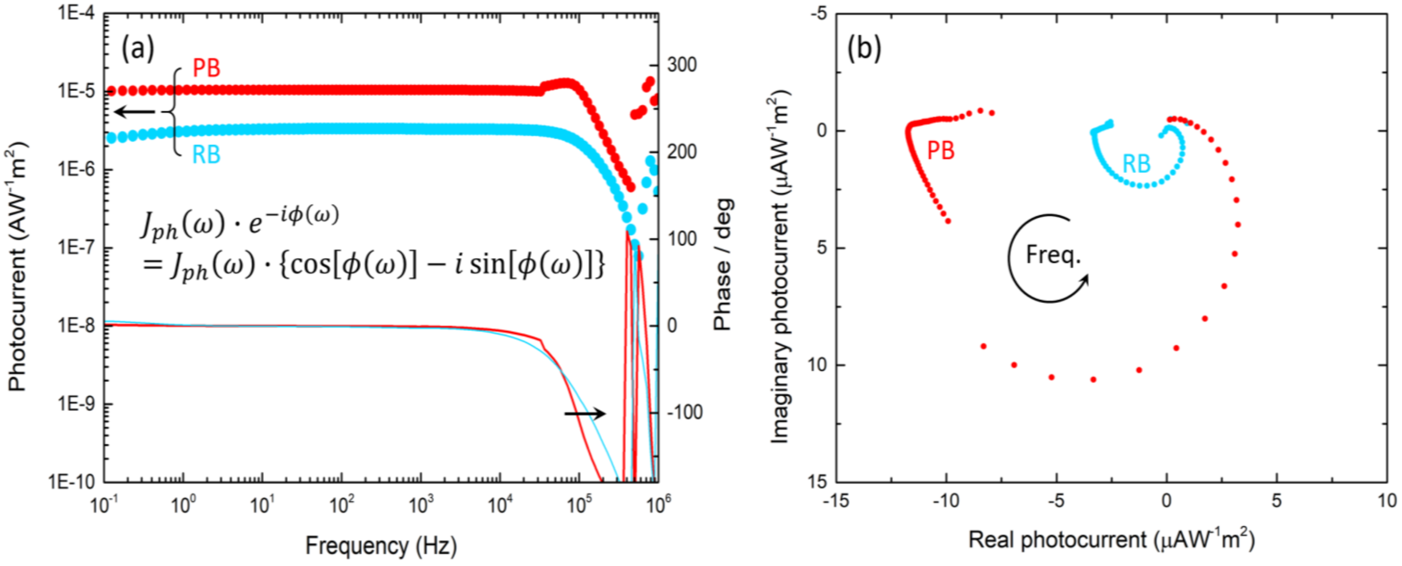

Supplementary Figure 6 – Intensity-modulated photocurrent spectroscopy (IMPS) analyses, IMPS analysis of CIS-PB (red) and CIS-RB (cyan) films expressed as Bode **(a)** and Nyquist plots **(b)**. The photocurrent appears as dots, while the phase angle is shown by the solid lines in (a).

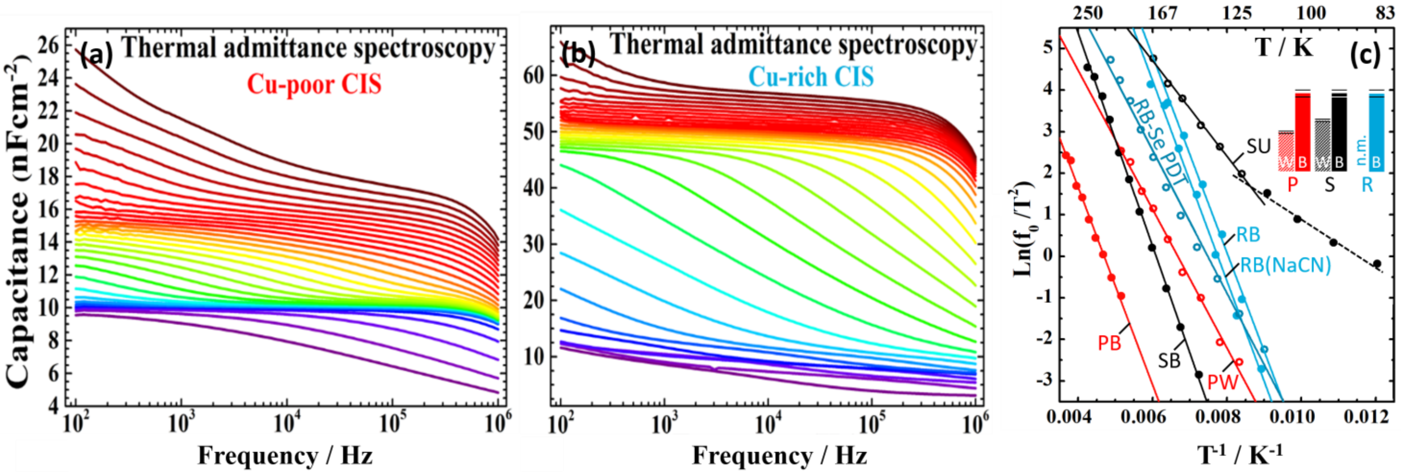

Supplementary Figure 7 – Thermal admittance analyses. Thermal admittance spectra for temperatures between 320 K and 50 K of a typical CIS-PW or CIS-PB device (CIS-PW is shown) **(a)** and CIS-RB device **(b)**. **(c)** Arrhenius plots obtained from the admittance spectra of devices comprising CIS-P (red), CIS-S (black) and CIS-R (blue) films. The samples yield activation energies of 200$\pm$20 meV if subjected to KCN-B etching (full dots) or lower (hollow dots) in all other cases.

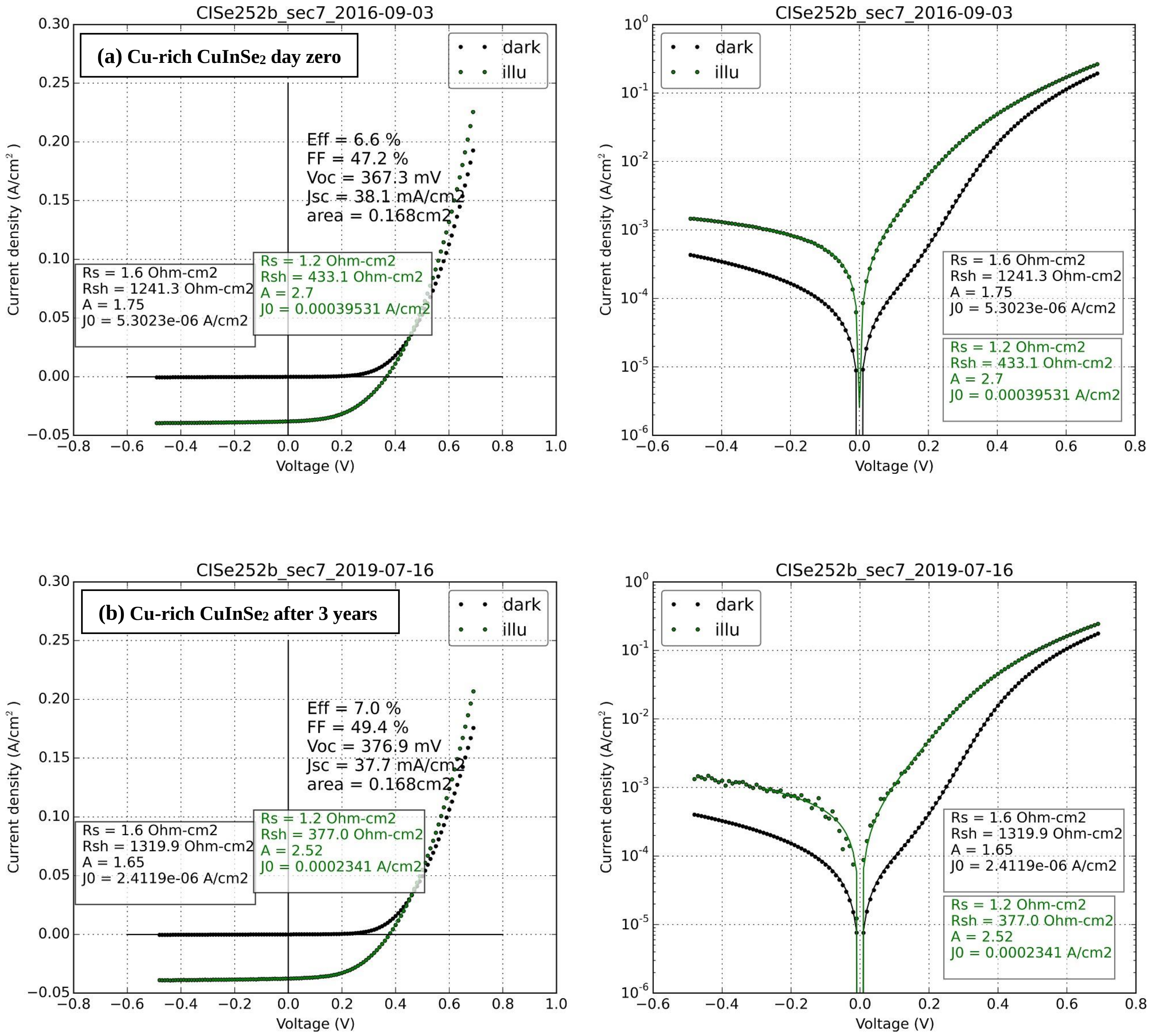

Supplementary Figure 8 – Ageing effect of Cu-rich CIS devices on current-voltage (J-V) analyses. Device J-V curves of Cu-rich $CuInSe_2$ **(a)** on day zero **(b)** after 3 years storage in vacuum; the two curves show no significant change in device characteristic with time under vacuum.

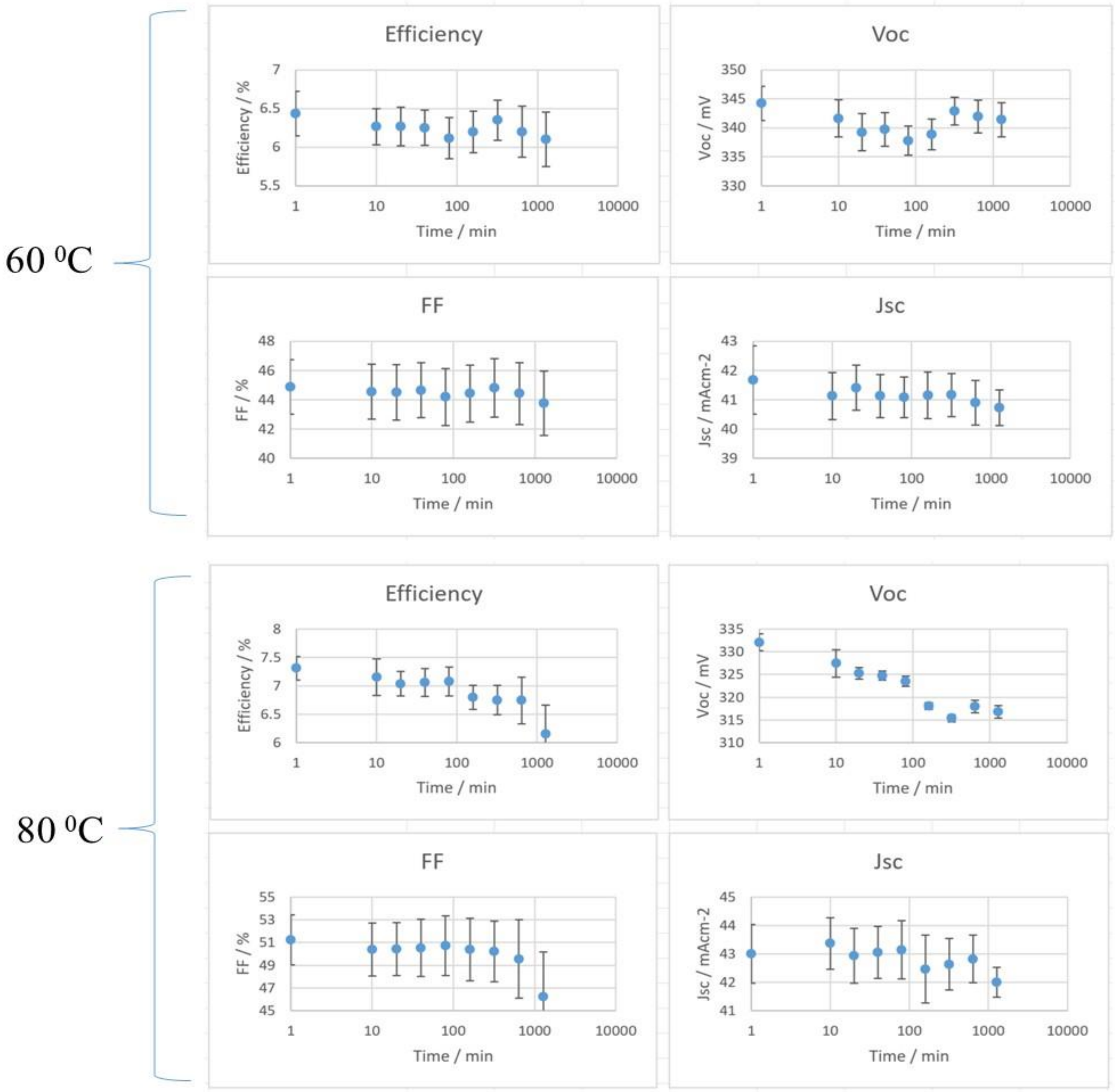

Supplementary Figure 9 – Low-temperature annealing of Cu-rich CIS devices on current-voltage (J-V) analyses. J-V characteristics of Cu-rich $CuInSe_2$ 60 ^{0}C and 80 ^{0}C-annealed devices as a function of annealing time.

Supplementary Note 1 – Oxidation of CIS bulk and surfaces

Thermodynamic driving forces do not provide information on reaction rates. Here, the oxidation rate of In_2Se_3 is first assessed in order to corroborate the thermochemical data in Figure 3a at room temperature. In_2Se_3 comes in different polymorphs, some of which are two-dimensional (2D) structures with fundamental and technological appeal as candidates in multi-layer van der Waals (vdW) heterostructures [3,4]. Besides being relevant for the vdW research field, knowing the oxidation rate of a 2D In_2Se_3 where the crystallographic planes are electronically isolated can give a lower estimate for the oxidation rate in (3D) $CuInSe_2$. Thus, 10 nm of β-In_2Se_3 (equivalent to 10 quintuple-layers, QL) were grown by molecular beam epitaxy (MBE) on sapphire and analysed by Raman spectroscopy. Although the intent was to assess the oxidation in air under dark conditions, it turned out that the compound oxidises while being analysed under the laser beam. Supplementary Figure 3a shows a 6-fold decrease of the A_1 interlayer vibrational mode over less than 5 minutes of exposure to air under laser illumination, suggesting that during this timeframe the majority of the 10 original In_2Se_3 QL have likely incurred transformation. Epitaxial CIS films on GaAs were subjected to different oxidation treatments in order to assess if the Gibbs free energy shown in Figure 3a translates into appreciable rates for the ternary compound. The epitaxial nature of the samples allows to exclude any effect of the grain boundaries on CIS oxidation, providing a proxy for the behaviour of grain surfaces. The first sample was oxidised at room temperature for 2 hours (*RT-ox*), the second one at 570 ^{0}C for 30 minutes in a tube furnace kept at 10^{-2} mbar of air in the presence of an elemental selenium source (*HT-Se*) and the third one was subject to the same treatment of *HT-Se* but in the absence of selenium (*HT*).

Supplementary Figure 3 summarizes the experimental results. The microstructure of *RT* is undistinguishable from that of a typical epitaxial film, while *HT-Se* and *HT* samples show clear signs of phase outgrowth on the CIS surface. EDS analysis at 7 keV suggests that In oxides have grown on the surface of *HT-Se* and *HT*, as the In/Cu atomic ratio increases up to *ca.* 3, accompanied by an increase of the O/(O+Se) ratio up to *ca.* 0.7. The photoluminescence (PL) yield is negatively affected by the oxidation treatments. The PL maximum intensity decreases with the increase of the In/Cu and O/(O+Se) ratios. In all cases, etching with KCN does not seem to alter the surface microstructure; however, the In/Cu EDS ratio of the *RT* film increases, while the O/(O+Se) EDS ratio decreases. This is consistent with the removal of Cu-Se and In-O phases by KCN.

Conversely, the surface composition of *HT-Se* and *HT* films seems unaffected by the etching treatment, suggesting that KCN is unable to remove the outgrown phases if the samples are oxidised at high temperature. Cu_2Se cannot be distinguished from $CuInSe_2$ by XPS, but the EDS measurements of the *RT* film are consistent with Cu-Se phase formation as a result of oxidation and its selective removal by the etchant. The results suggest that CIS oxidation occurs appreciably at room temperature, following reaction (02); importantly, the oxidation cannot be perceived morphologically. The spontaneous oxidation of CI(G)S in air is noteworthy, because it provides a chemical explanation for the much needed KCN treatment before the buffer deposition: etching is not needed just to remove residual Cu-Se phases formed during the growth, but to remove Cu-Se phases that form as soon as the absorber is exposed to air. Oxidation at high temperature is markedly different. Unlike in the *RT* case, KCN seems unable to remove In-O phases in the *HT* films. In order to clarify this point, the solubility of bulk In_2O_3 (Alfa Aesar, CAS 1312-43-2) in 5 wt. % KCN was assessed and estimated to be < 10^{-2} g/l. Therefore, the decreased O/(O+Se) ratio of the *RT* film upon etching must be a result of *mechanical* detachment of the In-O phase attached to the Cu-Se phases being *chemically* dissolved by KCN. Conversely, the In_2O_3 solubility in HCl 37 wt. % is comprised between $5\cdot10^{-2}$ g/l and 10^{-1} g/l.

It is concluded that no Cu-Se phases form on the surface of Cu-poor CIS at high temperature, or if such phases form they are readily “reabsorbed” through solid state reaction. This is consistent with the fast Cu redistribution in the Cu deficient CIS, as previously suggested, leaving only In-O phases on the CIS surface [5,6]. After the KCN etching, the photoluminescence yield increases the most for the *RT* film, i.e. the film from which most secondary phases are removed. In order to unambiguously link PL yield and film oxidation, the 15 mm library sample was fabricated and deliberately oxidised to yield the oxidation gradient along the length. The oxidation was carried out at 570 ^{0}C to ensure that no Cu-Se phases are formed, so any effect is limited to the presence of In_2O_3 phases or to any point defect associated with In_2O_3 formation. EDS and PL analyses are performed along the gradient and confirm the trend (Figure 3b).

These oxidation experiments suggest that the surface of CIS is extremely sensitive and prone to chemical modifications involving different point defects (Supplementary Figure 4) depending on the composition of the material. Likewise, it is not surprising to expect profound changes to CIS surface upon exposure to etching solutions, besides the traditional intended removal of secondary phases.

Supplementary Note 2 – Validity of (Cu-Se) double vacancy formation

The long time constants of the processes recorded by both PEC and SPV analyses are concluded to be consistent with the DFT model by Lany and Zunger [7], formulated precisely to address theoretically the light-induced metastabilities widely observed experimentally by other means.

The newer DFT results by Pohl and Albe [2] do not exclude the explanation of metastable behaviour by the Lany-Zunger model. They show that, in thermal equilibrium, the formation the Se vacancy is not favourable, but neither growth nor etching processes occur at equilibrium. Se gets leached from the absorber surface, as shown in ref. [8]. Furthermore, the conditions during etching are likely close to point D in the phase diagram of Pohl and Albe, where the formation energy of the Se vacancy is lowest. This is not to be confused with growth conditions, which are close to point A (for Cu-poor) and B (for Cu-rich). Selenium gets removed from the crystal during the etching process. Thus, the crystal has to form Se vacancies. There is experimental evidence that even in Cu-rich $CuInSe_2$ Cu vacancies exist (for a review see ref. [9]). Thus, it is very reasonable to assume that the double vacancy (Lany-Zunger type defect) forms.

Supplementary Note 3 – Stability of Cu-rich $CuInSe_2$ devices under vacuum and air

To test the stability of Cu-rich devices we perform two different experiment: First keeping the device under vacuum stored in desiccator for a long period with no air exposure and measuring J-V. Secondly, through accelerated oxidation by annealing the devices at 60 ^{0}C and 80 ^{0}C for up to 1280 minutes and measuring J-V at regular intervals.

J-V characteristics of Cu-rich $CuInSe_2$ devices do not show considerable change before (Supplementary Figure 8a) and after a long period of storage in the desiccator (Supplementary Figure 8a). The results suggest that Cu_2Se does not form spontaneously under vacuum.

Supplementary Figure 9 shows the results for the accelerated oxidation study. Clearly, there is no considerable change in the J-V characteristics of device annealed at 60 ^{0}C. However, the device annealed at 80 ^{0}C shows slight degradation in all the device parameters specially after 80 minutes of annealing. This latter effect may be a consequence of Cu_2Se phase formation by oxidation according to equation (05).

Supplementary references

1. Malitckaya, M., Komsa, H.-P., Havu, V. & Puska, M. J. First-Principles Modeling of Point Defects and Complexes in Thin-Film Solar-Cell Absorber $CuInSe_2$. *Advanced Electronic Materials* **3**, 1600353 (2017).
2. Pohl, J. & Albe, K. Intrinsic point defects in CuInSe2 and $CuGaSe_2$ as seen via screened-exchange hybrid density functional theory. *Phys. Rev. B* **87**, 245203 (2013).
3. Geim, A. K. & Grigorieva, I. V. Van der Waals heterostructures. *Nature* **499**, 419–425 (2013).
4. Balakrishnan, N. *et al.* Epitaxial growth of gamma-InSe and alpha, beta, and gamma-In_2Se_3 on epsilon-GaSe. *2D Mater.* **5**, 035026 (2018).
5. Rau, U. *et al.* Oxygenation and air-annealing effects on the electronic properties of $Cu(In,Ga)Se_2$ films and devices. *Journal of Applied Physics* **86**, 497–505 (1999).
6. Guillén, C. & Herrero, J. Recrystallization and components redistribution processes in electrodeposited $CuInSe_2$ thin films. *Thin Solid Films* **387**, 57–59 (2001).
7. Lany, S. & Zunger, A. Light- and bias-induced metastabilities in $Cu(In,Ga)Se_2$ based solar cells caused by the (V_{Se}-V_{Cu}) vacancy complex. *Journal of Applied Physics* **100**, 113725 (2006).
8. Elanzeery, H. *et al.* Challenge in Cu-rich $CuInSe_2$ thin film solar cells: Defect caused by etching. *Physical Review Materials* **3**, 055403 (2019).
9. Spindler, C. *et al.* Electronic Defects in $Cu(In,Ga)Se_2$: Towards a Comprehensive Model. *Phys. Rev. Materials* **3**, 090302 (2019).
